# Polymeric Sealing Materials in Pneumatic Actuators: From Degradation Mechanisms to Failure Diagnostics and Service-Life Prediction

**DOI:** 10.3390/ma19194213

**Published:** 2026-10-02

**Authors:** Marta Żyłka

**Affiliations:** Department of Aerospace Engineering, The Faculty of Mechanical Engineering and Aeronautics, Rzeszow University of Technology, Av. Powstancow Warszawy 12, 35-029 Rzeszow, Poland; mzylka@prz.edu.pl

**Keywords:** polymeric seals, pneumatic actuators, polyurethane, nitrile rubber, fluoroelastomers, degradation, tribology, leakage, diagnostics, service-life prediction

## Abstract

Polymeric sealing materials play a critical role in the reliability, efficiency, and dynamic performance of pneumatic actuators. Their long-term behavior is governed by the interaction between polymer structure, operating conditions, degradation mechanisms, tribological damage, and loss of sealing functionality. This review compares the principal sealing materials used or considered for pneumatic systems, including polyurethane (PU), thermoplastic polyurethane (TPU), nitrile butadiene rubber (NBR), hydrogenated nitrile butadiene rubber (HNBR), fluoroelastomers (FKM), ethylene–propylene–diene rubber (EPDM), silicone elastomers, and PTFE-based materials. Particular attention is given to thermo-oxidative aging, hydrolysis, crosslinking, chain scission, swelling, compression set, fatigue, and wear. Their selection involves trade-offs between friction, wear resistance, chemical and thermal stability, elastic recovery, compression set, and leakage performance. Material-characterization and condition-assessment methods, including scanning electron microscopy/energy-dispersive X-ray spectroscopy (SEM/EDS), Fourier-transform infrared spectroscopy (FTIR), electron paramagnetic resonance (EPR), thermal analysis, mechanical testing, and tribological measurements, are evaluated with respect to their ability to detect material deterioration. Accelerated-aging and service-life prediction approaches are also discussed. The literature shows that no single material is optimal under all pneumatic operating conditions and that material degradation does not necessarily coincide with functional failure. Future research should therefore integrate physicochemical diagnostics with friction, leakage, and actuator-performance measurements to develop reliable degradation-state and remaining-life models for pneumatic sealing systems. This review compares the principal sealing materials used in pneumatic systems, including PU/TPU, NBR, HNBR, FKM, EPDM, silicone elastomers, and PTFE-based materials.

## 1. Introduction

Pneumatic systems are an important actuation technology in modern manufacturing and automation. Pneumatic actuators are widely used in industrial automation, robotics, transportation systems, and medical and rehabilitation devices because of their simple construction, low weight, rapid response, and suitability for repetitive motion. Their reliability depends strongly on the performance of dynamic sealing systems, which determine leakage, friction, motion repeatability, energy efficiency, and long-term operating stability [1,2,3]. In reciprocating pneumatic cylinders, seal degradation may progressively increase friction and leakage and may ultimately become one of the factors limiting actuator service life [2,3].

Polymeric and elastomeric materials are particularly suitable for sealing applications because they combine deformability, elastic recovery, chemical resistance, and relatively low friction. The most relevant material classes include polyurethane and thermoplastic polyurethane, nitrile butadiene rubber, hydrogenated nitrile butadiene rubber, fluoroelastomers, ethylene–propylene–diene rubber, silicone elastomers, and polytetrafluoroethylene-based materials [1]. Their suitability depends not only on initial mechanical properties but also on their ability to retain elasticity, surface integrity, and sealing pressure during long-term exposure to cyclic deformation, frictional heating, compressed air, moisture, lubricants, contaminants, and temperature variations [1,4,5,6,7].

Previous reviews have discussed polymeric sealing materials mainly in the context of hydraulic actuators. Skowrońska et al. systematically reviewed materials used in hydraulic cylinders and sealing systems and showed that polymeric seals are selected primarily because of their favorable frictional behavior and ability to interact effectively with the mating surface [1]. However, pneumatic and hydraulic sealing conditions are not equivalent. Pneumatic systems operate with a low-viscosity gaseous medium, often under limited lubrication, and are therefore particularly sensitive to dry or boundary friction, surface condition, leakage, and environmental humidity. Consequently, conclusions derived from hydraulic seals cannot always be transferred directly to pneumatic actuators.

Experimental studies focused specifically on pneumatic seals remain relatively limited. Mazza and Goti demonstrated that the durability of reciprocating lip seals under dry pneumatic conditions depends strongly on material, geometry, and alignment, and that polyurethane can provide markedly better wear resistance than NBR under selected operating conditions [2]. Other pneumatic-cylinder investigations have likewise shown that seal selection substantially affects actuator lifetime and reliability [3]. These results emphasize that polymer type should not be considered independently of contact mechanics and actual operating conditions.

Further pneumatic-specific studies have demonstrated that seal friction and durability are strongly influenced by individual sealing elements, guide-bearing design, and seal geometry. Mazza and Belforte analyzed the contribution of individual piston and rod seals to the total friction force in pneumatic actuators [8]. Ambu et al. showed that guide-bearing redesign and redistribution of contact pressure can reduce wear and improve pneumatic-cylinder service life [9]. Belforte et al. developed a low-friction multi-lobed seal and demonstrated that optimization of seal geometry and lubrication conditions can improve friction and wear behavior [10].

The degradation of sealing polymers is a multiscale process. At the molecular level, thermo-oxidative aging, hydrolysis, chain scission, additional crosslinking, swelling, and additive migration can progressively modify the polymer network [4,5,7,8,9,10,11]. These changes affect hardness, stiffness, elastic recovery, compression set, and viscoelastic behavior and may subsequently alter the tribological response of the seal. At the surface scale, degradation can manifest as roughening, microporosity, cracking, abrasion, material transfer, debris formation, or local loss of conformity with the counterface [4,7,11]. The final functional consequences include increased friction, unstable motion, reduced contact pressure, and leakage.

The importance of coupling chemical aging with tribological behavior is particularly evident for NBR. Hydrothermal aging has been shown to increase crosslink density, promote additive loss and surface micropore formation, and degrade both elasticity and tribological performance [4]. These effects are directly relevant to pneumatic systems because residual humidity and condensate may be present in compressed-air installations. Similarly, studies of HNBR exposed to elevated temperature, oil, and compression demonstrate that aging involves simultaneous changes in chemical structure, crosslink density, compression set, mechanical behavior, and fracture morphology [5]. Such results indicate that seal degradation cannot be adequately characterized by a single macroscopic parameter.

Long-term elastomer-aging studies further demonstrate that measurable material degradation does not necessarily coincide with immediate loss of sealing capability. Kömmling et al. showed that EPDM and HNBR O-rings may remain leak-tight even after substantial deterioration of material properties [6]. Five-year aging experiments on HNBR, EPDM, and FKM also demonstrated significant differences between short-term accelerated-aging predictions and long-term behavior and highlighted the importance of compression set and appropriate end-of-life criteria [7]. These findings are important for pneumatic seals because material degradation and functional failure should be treated as related but distinct phenomena.

Fluoroelastomers represent a contrasting case because their fluorinated backbone provides comparatively high thermo-oxidative and chemical stability. Nevertheless, FKM is also susceptible to degradation under severe thermal conditions, and aged surfaces can become more vulnerable to abrasive damage [11]. Therefore, even chemically resistant elastomers must be assessed in relation to the dominant combination of temperature, friction, contaminants, and deformation.

Reliable characterization of sealing degradation requires complementary analytical methods. Conventional mechanical tests such as hardness, tensile testing, compression set, and stress relaxation provide information on the evolution of bulk properties, whereas scanning electron microscopy (SEM) and profilometry reveal surface damage and wear morphology. Fourier-transform infrared spectroscopy (FTIR), thermal analysis, and dynamic mechanical analysis can identify chemical and viscoelastic changes associated with aging [4,5,6,7]. More recently, electron paramagnetic resonance (EPR) combined with SEM-EDS has been applied to polyurethane seals removed from pneumatic drives, allowing for service-induced changes in paramagnetic centers and microstructure to be identified [12]. This demonstrates the potential of combining spectroscopic and microscopic information with functional seal assessment.

Lifetime prediction represents a further challenge. Accelerated aging is widely used as an experimental methodology for polymer durability assessment, whereas Arrhenius-based extrapolation, time–temperature superposition, and kinetic modelling are applied to interpret accelerated-aging data and estimate long-term behavior [13]. However, prediction accuracy depends on whether accelerated testing reproduces the degradation mechanisms occurring under actual service conditions. Long-term O-ring experiments have shown that extrapolation from short aging periods can be unreliable when degradation kinetics change with temperature or when compression and diffusion effects are neglected [7].

Despite the available knowledge, the literature remains fragmented. General reviews address polymer degradation and lifetime prediction [13], actuator-material reviews focus predominantly on hydraulic systems [1], and pneumatic-specific studies usually investigate individual seal materials, particular wear conditions, or selected diagnostic methods [2,3,4,12]. A comprehensive synthesis linking polymer chemistry and structure, pneumatic operating stressors, degradation mechanisms, tribological damage, leakage, diagnostic indicators, and service-life prediction has not yet been established specifically for pneumatic actuator sealing systems.

Accordingly, the aim of this review is to critically examine polymeric sealing materials used in pneumatic actuators from a materials-science perspective. The review compares the principal material classes, evaluates their dominant degradation and failure mechanisms, discusses relevant characterization techniques, and assesses approaches for accelerated aging and lifetime prediction. Particular emphasis is placed on identifying the relationships between material degradation and functional sealing performance. On this basis, an integrated framework for material selection, degradation diagnostics, and service-life assessment is proposed, together with research directions for more reliable pneumatic sealing systems.

Literature Search Strategy. The literature considered in this review was identified using major scientific databases, including Scopus, Web of Science, and ScienceDirect. Searches were performed using combinations of keywords related to pneumatic actuators, polymeric seals, polyurethane, NBR, HNBR, FKM, EPDM, silicone, PTFE, degradation, aging, friction, wear, leakage, diagnostics, and service-life prediction. Priority was given to peer-reviewed journal articles directly addressing polymeric sealing materials, degradation mechanisms, tribological behavior, diagnostic methods, and lifetime assessment. Studies concerning hydraulic, static, and other sealing systems were included where their findings were relevant to the interpretation of pneumatic-seal behavior.

**Figure 1:**
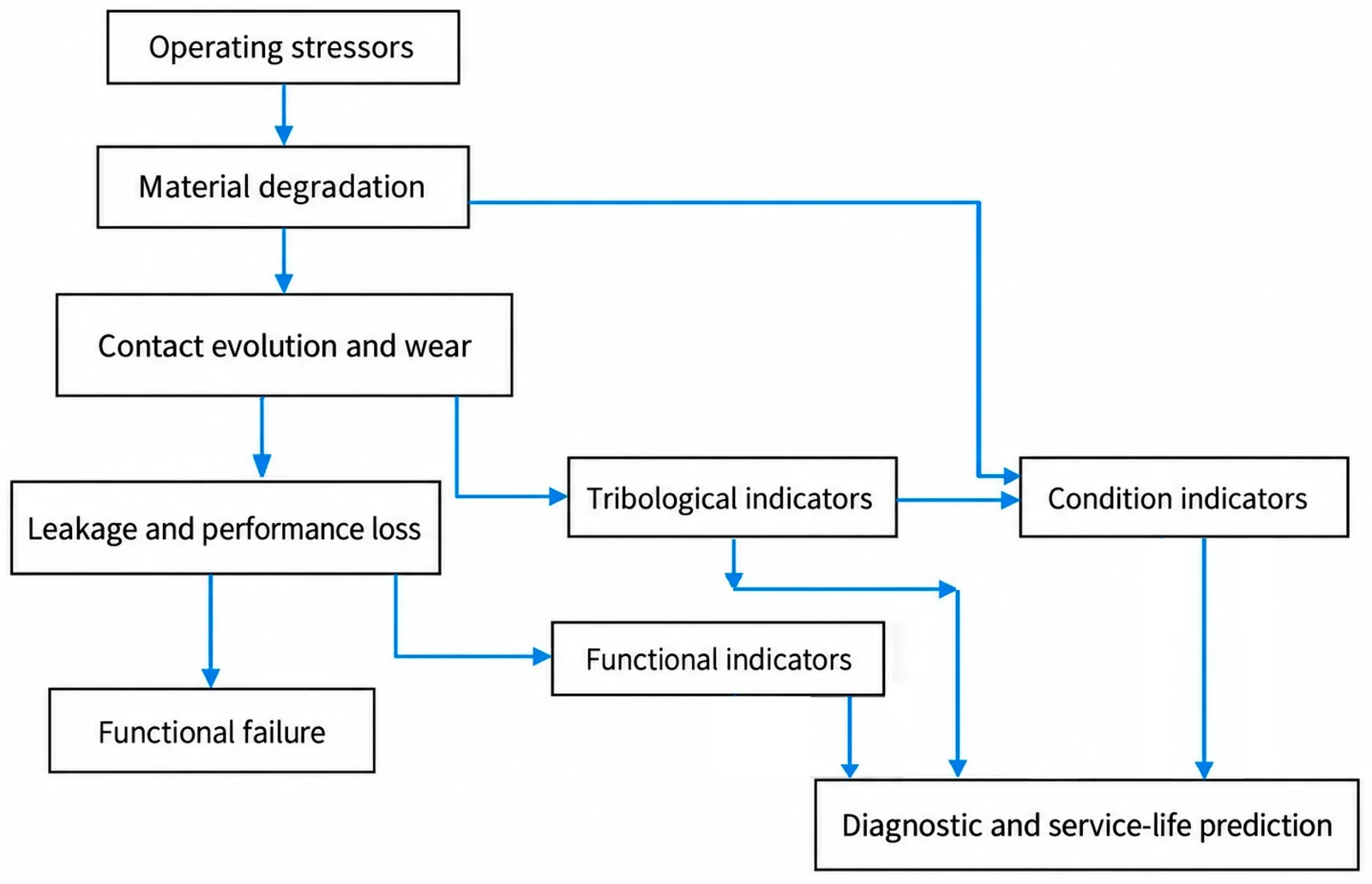
Conceptual pathway of polymeric seal degradation in pneumatic actuators.

To provide a conceptual overview of the reviewed problem, Figure 1 presents the relationships between operating stressors, material degradation, tribological deterioration, leakage, and functional failure in pneumatic sealing systems. The framework represents a general degradation pathway rather than a strictly sequential process, since several mechanisms may occur simultaneously and interact with one another. Diagnostic and condition-assessment indicators obtained at different stages of this pathway can support remaining-service-life prediction before functional failure occurs.

Previous pneumatic-specific studies have shown that seal geometry, guide-bearing design, and the contribution of individual sealing elements strongly affect friction, wear, and durability in pneumatic actuators. Analytical-experimental studies of seal friction, guide-bearing redesign, and low-friction multi-lobed seal concepts have confirmed the importance of tribological optimization in these systems [8,9,10]. However, these works address selected issues and do not provide a comprehensive materials-oriented synthesis linking polymer classes, degradation mechanisms, diagnostics, and service-life prediction [8,9,10].

## 2. Polymeric Sealing Materials for Pneumatic Actuators

The performance of a pneumatic seal is governed not only by its initial material properties but also by the stability of the polymer under repeated deformation, sliding contact, temperature changes, moisture, lubricant exposure, and contamination. Consequently, material selection should be based on the dominant service stressors rather than on a single parameter such as hardness or nominal temperature range. Polyurethane-based materials, NBR and HNBR, FKM, EPDM, silicone elastomers, and PTFE-based compounds represent the principal polymer classes relevant to pneumatic sealing systems [1,2].

The long-term performance of polymeric sealing materials is strongly governed by polymer chemistry and molecular architecture. Relevant structural factors include backbone composition, degree of saturation, chain polarity, segmented or crosslinked structure, crosslink density, filler content, and cure chemistry [1,4,5,11]. These features influence thermal and chemical stability, elastic recovery, friction, wear resistance, and susceptibility to aging [2,8,13,14]. Consequently, the behavior of sealing materials cannot be interpreted solely on the basis of polymer class, since differences in formulation and network structure may substantially modify degradation pathways and functional performance [4,5,14,15].

These materials exhibit distinctly different degradation pathways. PU generally provides high resistance to abrasion but may be susceptible to hydrolysis and friction-induced thermal degradation. NBR offers favorable oil resistance and cost efficiency but is more sensitive to thermo-oxidative aging, while HNBR improves thermal and oxidative stability [4,5]. FKM is preferred in chemically or thermally demanding environments but may progressively lose elastic recovery under severe aging conditions [6,7,8,9,10,11]. EPDM provides excellent resistance to water, ozone, and weathering, whereas silicone elastomers retain flexibility over a broad temperature range but generally exhibit lower wear resistance. PTFE and its composites provide low friction and high chemical inertness, although creep and wear must be controlled by material formulation and filler selection [1].

The following sections therefore compare these material groups with emphasis on the relationship between polymer structure, degradation mechanism, tribological response, and sealing functionality.

### 2.1. Polyurethane and Thermoplastic Polyurethane

Polyurethane (PU) and thermoplastic polyurethane (TPU) are widely used in dynamic sealing systems because they combine high abrasion resistance, load-bearing capability, elastic recovery, and favorable resistance to repeated mechanical loading [1,2]. Their properties arise from a segmented molecular structure containing relatively flexible soft segments and harder urethane-rich domains. The balance between these phases governs stiffness, resilience, damping, tensile behavior, and wear resistance and may differ substantially among individual PU formulations.

The relative proportion and chemistry of soft and hard segments, together with the degree of phase separation and crosslinking, strongly influence hydrolysis resistance, thermal stability, elastic recovery, and tribological performance [15].

For pneumatic applications, one of the principal advantages of PU is its resistance to reciprocating wear. Mazza and Goti showed that polyurethane seals used in pneumatic cylinders can exhibit markedly greater endurance than NBR under dry sliding conditions, although lifetime also depends strongly on seal geometry and axial alignment [2]. The result is important because it demonstrates that high intrinsic wear resistance does not eliminate the influence of contact mechanics.

Long-term PU degradation is governed by several interacting mechanisms. Frictional heating can modify hydrogen bonding and segmental mobility, while repeated deformation promotes fatigue and surface damage. Depending on formulation, moisture can initiate hydrolytic cleavage, particularly in polyester-based polyurethane systems. These processes may progressively reduce mechanical integrity and increase susceptibility to cracking, abrasion, and loss of sealing conformity [15].

An important observation for pneumatic systems is that chemical and tribological degradation do not occur independently. Friction generates heat, which accelerates physicochemical aging, while aging modifies stiffness and surface properties and thereby changes subsequent friction and wear. This feedback mechanism means that the long-term behavior of PU cannot be predicted from short-term friction or tensile tests alone.

Service-induced degradation has also been demonstrated directly in polyurethane seals removed from pneumatic drives. Żyłka et al. combined electron paramagnetic resonance spectroscopy with SEM-EDS and identified changes in paramagnetic centers together with distinct microstructural deterioration [12]. These results indicate that polymer degradation may be detected at the spectroscopic and microscopic levels before complete functional failure occurs.

The diagnostic value of combining analytical methods is important. SEM can reveal grooves, cracking, debris, and local material loss, while FTIR can identify changes in urethane bonds, oxidation, or hydrolysis. EPR provides complementary information on radical and paramagnetic species associated with degradation processes [12]. Mechanical tests such as hardness and compression set remain useful, but their interpretation should be linked to actual changes in friction and leakage.

PU performance is also strongly formulation dependent. Polyester- and polyether-based materials may differ significantly in hydrolytic resistance, while hard-segment fraction, additives, fillers, and processing conditions affect friction, elasticity, and durability. Therefore, studies reporting only “polyurethane” without detailed material characterization have limited value for lifetime prediction.

Overall, PU remains one of the strongest candidates for dynamic pneumatic sealing where abrasion resistance and reciprocating durability dominate. Its principal limitation is not inadequate initial performance, but the possibility that hydrolysis, frictional heating, contamination, and cyclic fatigue progressively reduce this advantage. Future pneumatic-seal studies should therefore correlate molecular and microstructural degradation with friction evolution, leakage, and remaining service life, rather than evaluating these phenomena separately.

### 2.2. Nitrile Butadiene Rubber and Hydrogenated Nitrile Butadiene Rubber

Nitrile butadiene rubber (NBR) is widely used in sealing applications because it combines favorable oil resistance, elastic recovery, moderate gas permeability, and relatively low cost. Its performance is strongly affected by acrylonitrile content, crosslink density, filler system, plasticizers, and curing chemistry. These formulation variables influence not only initial mechanical properties but also the rate and mechanism of aging under thermal, chemical, and tribological loading [1,4]. NBR is a random copolymer of acrylonitrile and butadiene, and its properties are strongly influenced by the acrylonitrile content, which affects polarity, oil resistance, flexibility, and low-temperature behavior [4,16]. Commercial NBR grades typically contain approximately 18–50 wt.% acrylonitrile, and increasing acrylonitrile content generally improves oil and fuel resistance while reducing low-temperature flexibility [16].

A major limitation of NBR is its sensitivity to thermo-oxidative and hydrothermal degradation. Elevated temperature can promote additional crosslinking and chain scission, while exposure to oils may either suppress or accelerate aging depending on fluid composition and additive migration. Liu et al. showed that the surrounding lubricant significantly altered the thermal-aging pathway of NBR, demonstrating that the simple classification of NBR as an “oil-resistant” elastomer is insufficient for lifetime assessment [17]. The interaction between elastomer and lubricant must therefore be considered as a coupled material–environment process.

Hydrothermal aging is particularly relevant to pneumatic systems because compressed air may contain residual humidity or condensate. Huang et al. demonstrated that hydrothermal exposure of NBR seals caused additive volatilization, increased crosslink density, deteriorated surface quality, and reduced elastic deformability. These changes were accompanied by worsening tribological performance, while surface micropores formed during aging were identified as an important contributor to increased friction [4].

This relationship between aging and tribology is important because pneumatic-seal failure does not necessarily begin with visible cracking or gross leakage. Progressive changes in surface morphology and elasticity can increase dynamic friction and modify contact pressure before catastrophic failure occurs. In precision pneumatic actuators, such changes may influence breakaway force, low-speed motion, positioning repeatability, and energy consumption even while the seal remains nominally tight [4].

Hydrogenated nitrile butadiene rubber (HNBR) provides improved thermal and oxidative stability because hydrogenation reduces the number of unsaturated bonds in the polymer backbone. It is therefore attractive for more demanding sealing environments. However, HNBR is still subject to significant degradation when temperature, fluid exposure, and compressive stress act simultaneously. HNBR is obtained by hydrogenation of the unsaturated butadiene units in NBR, which reduces backbone unsaturation and improves resistance to thermo-oxidative degradation while retaining the polarity associated with nitrile groups [5]. Commercial HNBR grades can differ substantially in residual unsaturation. Fully hydrogenated grades may contain less than 1% residual double bonds, whereas partially hydrogenated grades may retain several percent residual unsaturation, depending on the polymer grade [18].

Lou et al. investigated HNBR O-rings under simulated servo-system conditions and showed that degradation was governed largely by increasing crosslink density during much of the aging process, whereas chain scission became more important at higher temperature and longer exposure times. Compression set increased with temperature and aging duration, and changes in chemical structure were accompanied by deterioration of mechanical properties and development of a hardened brittle surface layer, voids, and agglomerates [5].

These results also demonstrate the importance of mechanical constraint during aging. Seals operate under compression and interference rather than as unstressed polymer specimens, and the deformation state can influence diffusion, local stresses, and reaction kinetics. Consequently, accelerated aging performed on unloaded bulk specimens may not reproduce the degradation behavior of an actual O-ring or reciprocating lip seal [5].

The comparison between NBR and HNBR therefore cannot be reduced to a simple ranking. HNBR generally provides greater resistance to thermal and oxidative degradation, whereas NBR remains attractive for moderate-temperature applications where cost and oil compatibility dominate. In both cases, however, long-term behavior depends on the interaction of temperature, humidity, lubricant chemistry, compression, and sliding contact [4,5,17].

From a diagnostic perspective, the most informative assessment combines several techniques. FTIR can identify chemical changes associated with oxidation and network modification, SEM reveals surface deterioration and fracture morphology, while compression-set and crosslink-density measurements provide information on loss of elastic recovery [4,5]. Tribological testing is essential because changes in bulk properties do not necessarily translate directly into changes in friction or leakage.

The main research gap for nitrile-based pneumatic seals is the lack of long-term studies linking material degradation directly to functional actuator performance. Existing studies provide detailed information on hydrothermal aging, oil exposure, compression set, and chemical changes, but comparatively few correlate these indicators with pneumatic leakage, friction evolution, or remaining service life. Future investigations should therefore combine accelerated aging with representative pneumatic cycling and simultaneously monitor material state, friction, leakage, and actuator performance.

### 2.3. Fluoroelastomers

Fluoroelastomers (FKM) are used in sealing systems when resistance to elevated temperature, fuels, oils, and chemically aggressive environments is more important than low material cost or maximum low-temperature flexibility. Their comparatively high stability originates from the fluorinated polymer backbone and the strength of carbon–fluorine bonds. In practical terms, FKM generally offers better thermo-oxidative and chemical resistance than NBR, although its dynamic behavior still depends strongly on formulation, cure system, filler content, and operating conditions [1,11]. FKM elastomers contain a highly fluorinated carbon backbone, and the strong C–F bonds contribute to their high resistance to heat, oxidation, fuels, and many aggressive chemicals [11,14,19]. Commercial FKM grades typically contain approximately 66–70 wt.% fluorine, depending on copolymer or terpolymer composition; higher fluorine content is generally associated with increased chemical resistance [19].

The main degradation mechanisms of FKM at elevated temperature include dehydrofluorination, chain scission, oxidation of newly formed unsaturated structures, and progressive loss of elastic recovery. Vega Puga et al. showed that accelerated thermo-oxidative aging of FKM O-rings produced measurable changes in chemical structure, compression set, stress relaxation, hardness, and leakage [14]. Importantly, the study demonstrated that functional seal failure should not be inferred from a single material parameter. Compression set and stress relaxation become particularly meaningful when they are related to actual loss of sealing capability [14].

Thermal degradation also affects tribological behavior. Qin et al. reported that degraded FKM became more susceptible to three-body abrasive wear, with aged surfaces developing cavities capable of retaining hard particles at the contact interface [11]. This altered particle motion and intensified damage to both the elastomer and the counterface. Such findings are relevant to pneumatic seals because contamination can transform moderate sliding wear into a more severe abrasive regime, particularly when the polymer surface has already been weakened by thermal aging.

Compared with NBR, FKM generally exhibits greater resistance to multi-factor aging. Recent studies involving combinations of temperature, humidity, compression, and chemical exposure have shown more pronounced structural deterioration and compression-set growth in NBR than in FKM [20]. However, the superior chemical stability of FKM does not guarantee longer service life under all conditions. Dynamic seals also require adequate flexibility, low friction, and recovery after cyclic deformation, and these properties may still deteriorate during prolonged aging.

The principal advantage of FKM in pneumatic actuators is therefore its resistance to thermal and chemical stress, while its main limitation is the possibility of gradual hardening and loss of elastic recovery under severe or prolonged exposure. For this reason, FKM is most appropriate when the dominant failure risk is chemical or thermo-oxidative rather than purely abrasive.

From a diagnostic standpoint, the most informative assessment combines FTIR or infrared microscopy with compression-set testing, stress-relaxation measurements, hardness, SEM, and leakage evaluation [11,14]. Such multi-method characterization is particularly useful because chemical degradation, mechanical aging, and functional failure do not occur at the same rate.

A major gap in the current literature is the limited number of studies addressing FKM under realistic dynamic pneumatic conditions. Most detailed investigations concern static O-rings, high-temperature systems, or other demanding sealing applications. Future work should therefore couple moderate thermo-oxidative aging with reciprocating pneumatic cycling and monitor friction, leakage, compression recovery, and chemical degradation simultaneously.

### 2.4. EPDM and Silicone Elastomers

Ethylene–propylene–diene monomer rubber (EPDM) is used in sealing systems where resistance to water, steam, ozone, and environmental aging is required. Its largely saturated polymer backbone provides good thermo-oxidative stability compared with NBR, while its low compatibility with mineral oils limits its use in lubricated pneumatic systems [6,7]. Long-term studies of compressed EPDM O-rings have shown that aging is strongly influenced by temperature, compression, and diffusion-limited oxidation, which may lead to non-uniform property changes across the seal cross-section [21]. EPDM has a predominantly saturated ethylene–propylene backbone with a small amount of diene units that enable crosslinking, and this low level of backbone unsaturation contributes to its good resistance to ozone, weathering, and thermo-oxidative aging [21,22]. In commercial EPDM grades, the ethylidene norbornene (ENB) content is typically in the range of a few weight percent, with representative grades containing approximately 2.8–7.5 wt.% ENB; this parameter influences crosslinking behavior and final network properties [23].

Aging of EPDM is associated mainly with changes in crosslink density, hardness, compression set, and elastic recovery. Under combined thermal and compressive loading, crosslinking may dominate over chain scission, while prolonged compression can accelerate deterioration of sealing resilience [22]. These effects are particularly important for pneumatic seals because reduced recovery decreases the ability of the elastomer to maintain contact pressure after repeated deformation or temperature changes.

Silicone elastomers offer a different property profile. Their siloxane backbone provides excellent flexibility over a broad temperature range and makes them attractive for applications involving thermal cycling or low-temperature operation. However, silicone rubbers generally exhibit lower abrasion and tear resistance than PU, NBR, or HNBR, which restricts their use in heavily loaded reciprocating seals.

Silicone elastomers are characterized by a flexible Si–O–Si backbone, which gives the polymer high chain mobility and contributes to its broad service-temperature range and good low-temperature flexibility [24,25]. This high chain flexibility is reflected in a very low glass-transition temperature, typically close to −120 °C for silicone elastomers, which contributes to their ability to retain flexibility at low temperatures [26].

Thermo-oxidative aging of silicone sealing materials leads to increasing hardness and compression set, changes in surface roughness, and gradual deterioration of contact conditions [24]. Experimental and numerical studies have shown that these material changes reduce sealing performance even when macroscopic failure is not yet visible [24]. Long-term storage studies likewise indicate that silicone seals can undergo significant microstructural and mechanical degradation, detectable by SEM, FTIR, thermal analysis, and compression-set measurements [25].

From a materials-selection perspective, EPDM and silicone should therefore be treated as special-purpose sealing materials. EPDM is particularly attractive when humidity, water, ozone, or environmental exposure dominate, whereas silicone is advantageous where retention of flexibility over a wide temperature range is critical. Their principal limitation in conventional dynamic pneumatic actuators is that these environmental advantages do not necessarily translate into superior wear resistance.

The main research gap is the limited amount of pneumatic-specific endurance data for both material groups. Most available studies concern static O-rings or seals operating under thermal aging conditions. Direct comparisons of EPDM and silicone with PU, NBR, and FKM under realistic reciprocating pneumatic conditions—including friction, leakage, humidity, and cyclic loading—remain scarce.

### 2.5. PTFE and PTFE-Based Composites

Polytetrafluoroethylene (PTFE) occupies a distinct position among sealing polymers because its principal advantage is not high elasticity but very low friction, chemical inertness, and thermal stability. These properties make PTFE attractive for dynamic sealing systems in which friction losses must be minimized. However, unfilled PTFE exhibits relatively poor wear resistance and a pronounced tendency to creep, so practical seal grades are frequently modified with fillers such as carbon, graphite, glass fiber, bronze, PEEK, or other reinforcement phases [1,27,28].

In reciprocating sealing systems, PTFE performance depends strongly on the interaction between material composition, counterface topography, pressure, sliding velocity, and lubrication. Deaconescu and Deaconescu showed that both virgin and filled PTFE can provide favorable tribological behavior in coaxial hydraulic-cylinder seals, but the transition between lubrication regimes and the resulting friction depend on operating conditions and seal geometry [27]. Although hydraulic systems differ from pneumatic actuators, these results are relevant because they demonstrate that the low intrinsic friction of PTFE does not alone determine dynamic seal performance.

The major limitation of virgin PTFE is its high wear rate. Reinforcement can reduce wear substantially, sometimes by several orders of magnitude, although the improvement is often accompanied by changes in friction and counterface interaction [28]. Carbon- and glass-fiber-reinforced fluoropolymer composites have also been shown to improve sealing performance by promoting more stable transfer films and reducing leakage compared with unmodified PFA systems [29].

PTFE consists of a highly fluorinated –CF_2_–CF_2_– backbone, and the shielding effect of fluorine atoms together with the strong C–F bonds contributes to its chemical inertness, low surface energy, and low friction [27,28,30]. PTFE is a highly crystalline polymer, and its degree of crystallinity is commonly in the range of approximately 50–70%, depending on processing and thermal history; this strongly influences stiffness, creep resistance, and tribological behavior [31].

Transfer-film formation is therefore central to the tribological behavior of PTFE seals. During sliding, polymer fragments can be transferred to the metallic counterface, producing a thin tribofilm that modifies subsequent friction and wear. The stability of this film depends on filler type, surface chemistry, humidity, and counterface material. Recent work on PTFE composites showed that bronze-, PEEK-, and other filled systems can produce markedly different tribofilms, with corresponding differences in steady-state friction and wear [30].

Viscoelastic behavior also affects sealing performance. Reciprocating tests on filled PTFE composites have shown that friction and wear are sensitive to both material viscoelasticity and the ability of fillers to stabilize the transfer film on the counterface [32]. This is particularly relevant for pneumatic applications operating at low speed, where the transition between static and dynamic friction may influence stick–slip behavior and actuator repeatability.

For pneumatic actuators, PTFE-based materials may therefore be advantageous where low friction, chemical resistance, and reduced breakaway force are more important than the high elastic recovery provided by conventional elastomers. Their main limitations are creep, wear of unfilled grades, and sensitivity to counterface and filler selection. In practice, PTFE is often most effective as part of a composite or energized sealing system rather than as an unmodified material.

The principal research gap concerns the limited number of long-term studies on PTFE composites under realistic pneumatic conditions. Most detailed tribological investigations concern hydraulic, rotary, or valve seals [27,30,32]. Future pneumatic studies should therefore evaluate how filler type, transfer-film formation, humidity, low-speed reciprocation, and counterface roughness affect friction, leakage, and long-term dimensional stability.

### 2.6. Comparative Assessment of Sealing Materials

The comparison of polymeric sealing materials shows that no single material provides optimum performance under all pneumatic operating conditions. Material selection should therefore be based on the dominant combination of wear, temperature, humidity, lubricant exposure, chemical compatibility, friction requirements, and expected service life, rather than on a simple ranking of nominal material properties [1,2,4,11].

PU and TPU are particularly advantageous where abrasion resistance and reciprocating durability are dominant requirements. Their main limitations arise from formulation-dependent sensitivity to hydrolysis, frictional heating, and long-term physicochemical degradation [2,12,15]. NBR remains an economical and versatile sealing material with good oil resistance, but thermo-oxidative and hydrothermal aging can progressively increase crosslink density, reduce elasticity, and deteriorate tribological behavior [4,17]. HNBR provides improved resistance to thermal and oxidative degradation while retaining many of the functional advantages of NBR, although compression set and network changes remain important under prolonged thermo-mechanical loading [5].

FKM provides the greatest resistance to severe thermal and chemical environments among the principal elastomeric materials considered here. Its use is therefore justified when thermo-oxidative or chemical degradation dominates the expected failure process. However, progressive hardening, loss of elastic recovery, and increased susceptibility of degraded surfaces to abrasion can still limit functional lifetime [11,14,20]. EPDM is particularly suitable for humid, aqueous, ozone-rich, or outdoor environments, whereas silicone elastomers are attractive when flexibility over a broad temperature range is required. Their use in heavily loaded reciprocating contacts is more restricted because of compatibility or wear limitations [21,22,23,24,25,26].

PTFE and filled-PTFE systems differ fundamentally from conventional elastomers. Their main advantages are low friction, chemical inertness, and reduced breakaway resistance, while their limitations include creep and relatively poor wear resistance in unfilled grades. Composite reinforcement can greatly improve wear behavior, but performance depends strongly on filler type, counterface properties, and transfer-film formation [27,28,29,30,31,32].

A comparative overview of the principal polymeric sealing materials, including their main advantages, limitations, dominant degradation mechanisms, and application suitability, is presented in Table 1.

The ranges and qualitative assessments reported in this table were synthesized from the cited literature and may vary with polymer formulation, filler system, cure chemistry, and operating conditions.

The table also highlights an important difference between material resistance and functional seal durability. A polymer can possess excellent resistance to one degradation mechanism while remaining vulnerable to another. For example, FKM may outperform NBR under chemical and thermal loading but provide no advantage when low-temperature flexibility is the dominant requirement. Similarly, PU may provide superior abrasion resistance but lose this advantage under hydrolytic or severe thermal conditions. PTFE minimizes friction but requires appropriate reinforcement and seal design to control creep and wear [2,4,11,14,27].

The most appropriate material-selection strategy is therefore stressor-dependent. For conventional reciprocating pneumatic cylinders, PU and NBR/HNBR remain the most relevant general-purpose candidates. FKM becomes attractive when thermal or chemical demands increase, whereas EPDM and silicone serve more specialized environmental requirements. PTFE-based materials are particularly useful where friction reduction and chemical inertness outweigh the need for high intrinsic elasticity.

More importantly, the available literature indicates that initial material properties alone are insufficient for predicting service life. Long-term performance depends on how the material evolves under coupled exposure to cyclic deformation, friction, temperature, humidity, lubricants, and contamination [4,5,11,14]. This observation provides the basis for the next part of the review, which examines the operating stressors responsible for initiating and accelerating seal degradation.

## 3. Operating Stressors in Pneumatic Actuators

Polymeric seals in pneumatic actuators operate under a combination of mechanical, tribological, thermal, chemical, and environmental stresses. These factors rarely act independently. Instead, they interact and may accelerate degradation through coupled mechanisms. Frictional heating can intensify oxidation or hydrolysis, moisture can alter mechanical and tribological properties, and contamination can transform mild sliding wear into severe abrasion [4,11,15].

For this reason, the durability of a pneumatic seal should be considered as the response of a material–surface–environment system, rather than as an intrinsic property of the polymer alone. The most important stressors include reciprocating mechanical loading, friction and local heating, humidity, lubricant interaction, temperature, contamination, misalignment, and surface roughness.

### 3.1. Cyclic Mechanical Loading and Contact Stress

Piston and rod seals undergo repeated deformation during every actuator cycle. Local contact pressure is required to maintain tightness, but excessive interference or deformation increases friction and material stress. Repeated loading may promote fatigue, permanent deformation, and loss of elastic recovery, particularly when combined with elevated temperature or aging-induced hardening [2,5].

Seal geometry and alignment are therefore as important as polymer properties. Experimental studies on pneumatic cylinders have shown that even a durable material can fail prematurely when misalignment or unfavorable seating conditions modify the contact-pressure distribution [2]. In this sense, material degradation and mechanical design cannot be evaluated independently.

### 3.2. Friction and Frictional Heating

Friction at the seal–counterface interface is one of the most important stressors in dynamic pneumatic systems because it directly affects both actuator performance and material durability. Seal friction contributes to breakaway force, hysteresis, stick–slip behavior, and low-speed instability, while the generated heat may accelerate physicochemical degradation of the polymer [2,4,15].

Breakaway friction is governed by the transition from static to dynamic sliding and is therefore particularly important during actuator start-up and low-speed motion. Elevated breakaway force may result from high seal preload, insufficient lubrication, increased adhesion at the seal–counterface interface, surface roughness, or aging-induced changes in polymer viscoelasticity. When static friction substantially exceeds dynamic friction, repeated sticking and sudden release may occur, producing stick–slip motion. In pneumatic actuators, this behavior can cause velocity fluctuations, positioning errors, reduced repeatability, vibration, and deterioration of motion smoothness [2,33,34]. Mitigation strategies include appropriate seal geometry and preload, controlled lubrication, optimization of counterface roughness and surface texture, and the use of low-friction sealing materials or surface modifications [32,33,34,35,36]. Because frictional behavior changes with aging, breakaway force and low-speed stability may also serve as sensitive functional indicators of seal degradation before gross leakage develops.

The magnitude of friction depends on contact pressure, sliding velocity, counterface roughness, lubrication, seal geometry, and polymer viscoelasticity. Under dry or poorly lubricated conditions, repeated reciprocating motion can produce local temperature rises that exceed the bulk actuator temperature. This promotes softening or hardening, changes in segmental mobility, and progressive surface deterioration, depending on the polymer class [2,15].

For PU-based materials, frictional heating may weaken intermolecular interactions and increase susceptibility to abrasion or fatigue damage [14]. In NBR, aging-induced changes in crosslink density and surface morphology can modify frictional response [4]. In FKM, thermal degradation may increase surface damage and promote retention of abrasive particles within the contact interface [11].

These observations indicate that friction should not be treated only as a mechanical loss. It acts simultaneously as a functional parameter and a degradation driver. For this reason, future lifetime models should include the evolution of friction with aging rather than assuming constant tribological properties throughout service.

### 3.3. Humidity, Condensation, and Lubricant Exposure

Moisture is particularly relevant in pneumatic systems because compressed air may contain residual water vapor or condensate. Hydrothermal exposure can alter polymer structure, surface chemistry, and tribological behavior. NBR seals subjected to combined heat and humidity exhibited additive loss, increased crosslink density, reduced elastic deformability, and deterioration of surface quality and frictional performance [4].

PU may also be susceptible to hydrolysis, particularly polyester-based formulations, whereas EPDM generally provides better resistance to aqueous environments. Consequently, humidity may influence material ranking significantly and should be considered during seal selection rather than treated only as an air-quality issue.

Lubricants introduce another source of complexity. NBR is generally regarded as oil-resistant, but Liu et al. demonstrated that different lubricating oils can produce substantially different aging pathways because of additive extraction, diffusion, swelling, and oxidation processes [17]. Thus, nominal chemical compatibility does not guarantee long-term stability.

The effects of moisture and lubricants are particularly important because both can alter the polymer–counterface interface. Swelling, plasticizer migration, or surface softening may change contact pressure and friction even before significant bulk degradation occurs. Pneumatic-seal studies should therefore report air preparation, lubrication regime, and environmental humidity explicitly.

### 3.4. Contamination and Counterface Condition

Particles trapped within the seal–counterface interface can substantially accelerate wear. Contamination may originate from inadequate air filtration, corrosion products, assembly debris, degraded lubricant, or wear particles generated elsewhere in the actuator. Once introduced into the interface, particles can promote abrasive cutting, local indentation, surface scoring, and secondary debris formation [11].

The susceptibility to contamination depends on the degradation state of the polymer. Thermally aged FKM, for example, develops surface cavities that can retain abrasive particles and intensify three-body wear [11]. Similar interactions are relevant to PU and other relatively soft sealing polymers, in which embedded particles may act as local abrasive asperities.

Counterface topography is equally important. A surface that is too rough increases abrasive interaction, whereas an excessively smooth surface may impair lubricant retention and modify transfer-film formation. For PTFE-based systems in particular, tribological performance depends strongly on the formation and stability of the transfer film on the mating surface [27,28,30,32].

Counterface material is also an important tribological variable because its surface properties influence friction, transfer-film formation, and wear [27,28,29,30,31,32]. Different counterface materials and surface treatments may alter interfacial adhesion, transfer-film stability, and the resulting frictional response. Consequently, counterface material should be reported together with surface roughness and lubrication conditions when comparing sealing materials, particularly in PTFE-based systems in which transfer-film formation strongly affects tribological performance [27,28,29,30,31,32].

Accordingly, seal durability should be considered a property of the seal–counterface pair, not of the polymer in isolation.

### 3.5. Misalignment, Geometry, and Installation Effects

Material durability may be substantially reduced by unfavorable seal geometry or actuator misalignment. Experimental studies of pneumatic lip seals have shown that lifetime depends not only on polymer type but also on seat dimensions and axial alignment [2]. Misalignment produces non-uniform contact pressure and localized stress concentrations, which can accelerate wear even when the selected polymer possesses high nominal abrasion resistance.

This issue is particularly important when comparing materials experimentally. A higher wear rate should not automatically be attributed to inferior polymer properties unless geometry, preload, counterface roughness, and alignment are controlled. Otherwise, material and mechanical effects become difficult to separate.

Installation-induced damage represents a related problem. Local cuts, excessive stretching, twisting, or extrusion during assembly may create stress concentrators that later initiate cracks or leakage. Such defects can substantially shorten service life and may be incorrectly interpreted as material degradation.

Overall, mechanical design and installation quality determine the stress field experienced by the seal and therefore directly influence the rate at which material degradation becomes functional failure.

### 3.6. Coupled Stressors and Multi-Factor Degradation

The most important conclusion from the available literature is that pneumatic seals rarely degrade under a single isolated stressor. Temperature, friction, humidity, compression, contaminants, and lubricant exposure act simultaneously and may reinforce one another [4,5,11].

For example, frictional heating can accelerate oxidation; aging can increase stiffness and alter contact pressure; modified contact pressure can increase friction; and the resulting surface damage can trap contaminants and intensify abrasion. Similarly, moisture may change polymer chemistry while simultaneously affecting friction and elastic recovery.

This coupled behavior explains why accelerated-aging tests based solely on elevated temperature often provide incomplete lifetime predictions. A more representative approach should reproduce the main combinations of thermal, mechanical, tribological, and environmental loading expected in service. A summary of the principal operating stressors acting on polymeric seals in pneumatic actuators, together with their associated material responses and functional consequences, is presented in Table 2.

## 4. Degradation Mechanisms

Polymeric seal degradation results from changes in molecular structure, network architecture, morphology, and surface condition that accumulate during service. In pneumatic actuators, the most relevant mechanisms are thermo-oxidative aging, chain scission and crosslinking, hydrolysis, swelling and additive migration, compression set, fatigue, and tribological damage. These processes often occur simultaneously, and their interaction determines whether degradation remains detectable only at the material level or progresses to friction increase, leakage, and functional failure [4,5,11,17].

### 4.1. Thermo-Oxidative Aging, Chain Scission, and Crosslinking

Thermo-oxidative aging is one of the most common mechanisms affecting elastomeric seals. Elevated temperature accelerates oxidation of the polymer backbone and can promote both additional crosslinking and chain scission. The relative contribution of these reactions depends on polymer chemistry, temperature, oxygen availability, exposure time, and the surrounding medium [5,17].

In NBR and HNBR, increased crosslink density is frequently associated with higher hardness, reduced elongation, and loss of elastic recovery [4,5]. At more advanced stages, chain scission may become dominant and contribute to embrittlement and crack formation. FKM exhibits greater resistance to oxidation, but severe thermo-oxidative exposure can still lead to dehydrofluorination, backbone cleavage, and loss of sealing resilience [11,14].

The functional significance of these reactions lies in their effect on contact pressure. A seal that becomes progressively stiffer or less recoverable may still remain leak-tight temporarily, but its ability to compensate for dimensional changes and dynamic deformation is reduced. Therefore, thermo-oxidative degradation should be evaluated together with compression set, stress relaxation, friction, and leakage, rather than through hardness alone [6,7,14].

### 4.2. Hydrolysis and Moisture-Induced Degradation

Hydrolysis is particularly important for moisture-sensitive materials, especially selected polyurethane formulations. Water can attack hydrolysable bonds in the polymer structure, causing molecular-chain cleavage and progressive deterioration of mechanical properties. Polyester-based polyurethane is generally more susceptible than polyether-based formulations, although degradation rate also depends on temperature, humidity, and formulation.

In pneumatic systems, hydrolysis may be promoted by residual moisture or condensate in compressed air. NBR is not primarily hydrolytically degraded in the same way as polyester PU, but hydrothermal exposure can still modify crosslink density, promote additive loss, and deteriorate surface morphology and tribological behavior [4].

The key implication is that humidity acts as both a chemical and tribological stressor. Moisture-induced changes in elasticity and surface condition can alter friction and contact pressure before macroscopic damage becomes visible.

### 4.3. Swelling, Extraction, and Additive Migration

Interaction with lubricants and other fluids may cause swelling, extraction of plasticizers or antioxidants, or penetration of fluid components into the polymer network. These effects can alter dimensions, stiffness, friction, and chemical stability.

For NBR, Liu et al. demonstrated that thermal aging depends strongly on oil composition because additives can migrate between the lubricant and elastomer, changing the oxidation and crosslinking behavior [17]. Similar mechanisms are relevant to HNBR and other elastomers exposed to lubricated pneumatic environments.

Swelling is not necessarily irreversible, but dimensional recovery after desorption does not guarantee restoration of the original tribological state. Surface damage, altered crosslink density, or additive depletion may persist. This distinction is important because a seal may recover its nominal dimensions while retaining degraded frictional or mechanical behavior.

### 4.4. Compression Set and Stress Relaxation

Compression set describes the inability of an elastomer to recover its original geometry after prolonged deformation and is one of the most important functional aging indicators for seals. Stress relaxation reflects the gradual decrease in sealing force under constant deformation.

Long-term studies of HNBR, EPDM, and FKM O-rings have shown that these parameters evolve significantly with time and temperature and may be more closely related to sealability than tensile properties alone [6,7,14]. Nevertheless, neither compression set nor stress relaxation provides a universal end-of-life threshold because leakage also depends on seal geometry, interference, pressure, and mating-surface condition.

For pneumatic seals, the most useful approach is therefore to relate compression-set growth directly to functional parameters such as leakage rate, breakaway friction, and motion repeatability.

### 4.5. Fatigue, Cracking, and Surface Damage

Dynamic pneumatic seals are repeatedly deformed and slid against metallic surfaces, which creates conditions for fatigue damage. Repeated stress may initiate microcracks, promote surface tearing, and gradually remove material from the contact zone [2,15].

Fatigue often interacts with thermal and chemical aging. An aged polymer may become less compliant and therefore more susceptible to crack initiation, while repeated frictional heating may accelerate further physicochemical deterioration. This creates a feedback process in which mechanical fatigue and material aging reinforce each other.

Surface cracks are especially critical because they can develop into leakage pathways even when the bulk material retains substantial mechanical strength. Therefore, microscopic surface characterization should accompany conventional bulk tests when evaluating dynamic seal durability.

### 4.6. Tribological Wear and Coupled Degradation

Abrasive, adhesive, and fatigue wear are major contributors to functional seal deterioration. In PU, high abrasion resistance generally delays wear progression, but contamination, misalignment, and frictional heating can substantially reduce this advantage [2,15]. In aged FKM, surface cavities may retain hard particles and intensify three-body abrasion [11]. In PTFE-based systems, long-term behavior is strongly influenced by transfer-film stability and filler composition [27,28,30,32].

The most important conclusion is that tribological wear is rarely independent of chemical aging. Oxidation, hydrolysis, or swelling changes the surface response; friction modifies temperature; and wear exposes fresh polymer layers. Consequently, seal degradation should be regarded as a coupled physicochemical–tribological process rather than a sequence of isolated mechanisms. A comparative summary of the main degradation mechanisms, associated material changes, and their potential impact on seal performance is presented in Table 3.

## 5. Diagnostic and Characterization Methods

Most of the methods discussed in this section are laboratory-based material-characterization techniques, often applied to seals after removal from service. They are therefore used primarily for post-service condition assessment and identification of degradation indicators, rather than for continuous online fault diagnosis.

Reliable assessment of polymeric seal degradation requires methods capable of detecting changes at different structural levels. In this review, the term “diagnostics” is used primarily in the sense of post-service material characterization, condition assessment, and detection of degradation indicators, rather than exclusively as online or in-service fault diagnosis. No single technique is sufficient because molecular degradation, surface damage, bulk mechanical changes, and functional deterioration do not necessarily progress at the same rate. For pneumatic sealing materials, the most informative approach therefore combines spectroscopic, microscopic, mechanical, thermal, and tribological methods [4,5,6,7,11,12,14].

### 5.1. SEM and EDS

Scanning electron microscopy (SEM) is widely used to evaluate surface morphology, fracture features, cracks, pores, wear grooves, debris, and local material removal. In degraded NBR and HNBR, SEM has revealed microporosity, brittle regions, voids, and surface deterioration associated with aging [4,5]. In polyurethane pneumatic seals, SEM-EDS has additionally identified service-induced morphological changes and inorganic inclusions that can be related to wear or contamination [12].

EDS complements SEM by providing elemental information from selected surface regions. Although it does not directly characterize polymer-chain chemistry, it is useful for identifying metallic particles, fillers, or contaminants transferred from the counterface or surrounding components.

### 5.2. FTIR Spectroscopy

Fourier-transform infrared spectroscopy (FTIR) is one of the most useful techniques for monitoring chemical changes in sealing polymers. It can identify oxidation, changes in functional groups, hydrolysis, dehydrofluorination, and modifications of hydrogen bonding or polymer-network structure [5,15,17].

For NBR and HNBR, FTIR has been used to follow aging-induced changes associated with oxidation and crosslinking [5,17]. In FKM, infrared methods can reveal degradation pathways involving dehydrofluorination and subsequent oxidative modification [14]. For PU, FTIR is particularly useful for assessing changes in urethane-related bands and degradation associated with hydrolysis or thermal exposure [15].

More specific degradation indicators can be obtained from changes in characteristic absorption bands. Oxidative aging is commonly associated with an increase in carbonyl-containing species and may therefore be followed using changes in the carbonyl region or a carbonyl index referenced to a comparatively stable band. Hydrolytic degradation can lead to the formation of hydroxyl-containing products and corresponding changes in O–H absorption, together with modifications of ester- or urethane-related bands. In polyurethane materials, aging may alter the N–H stretching region, the urethane carbonyl band near 1700–1730 cm^−1^, and C–N or C–O–C related absorptions, reflecting changes in hydrogen bonding, urethane linkage stability, and soft-segment chemistry. Such spectral changes are most informative when evaluated relative to the unaged material rather than interpreted from a single band in isolation [15,37].

Its principal advantage is sensitivity to chemical transformation; its limitation is that chemical changes do not directly indicate the magnitude of friction or leakage.

### 5.3. Electron Paramagnetic Resonance

Electron paramagnetic resonance (EPR) provides information on paramagnetic centers and radical species that may develop during polymer degradation. Compared with SEM or FTIR, it is less commonly used in sealing diagnostics, but it can provide complementary information on degradation processes that are not visible through conventional mechanical testing.

Żyłka et al. demonstrated the usefulness of EPR for polyurethane seals removed from pneumatic drives, where changes in paramagnetic species were analyzed together with SEM-EDS observations [12]. The study showed that service-induced deterioration can be characterized at both spectroscopic and microstructural levels. In polyurethane seals, EPR spectra may contain contributions from carbon-centered radical species formed during polymer degradation, as well as signals associated with paramagnetic metal ions or metal-oxide inclusions, including Fe^3+^-related centers [12]. Spectral interpretation is based on parameters such as resonance-field position, g-factor, line width, signal intensity, and, where appropriate, temperature dependence. Changes in these parameters can indicate variations in the concentration and local environment of paramagnetic centers during service-induced degradation. In the study by Żyłka et al., spectral simulation supported the separation of contributions from carbon radicals, Fe^3+^ species, and metal-oxide-related centers, while SEM-EDS provided complementary information on the corresponding microstructural and elemental changes [12]. The main limitation of EPR is that its sensitivity depends on the type, concentration, and relaxation behavior of the paramagnetic species, as well as on instrumental settings and sample geometry. Consequently, a universal detection limit cannot be assigned to polymer-seal degradation, and low concentrations of short-lived or weakly paramagnetic species may remain undetected. EPR results should therefore be interpreted comparatively and in conjunction with complementary methods such as SEM-EDS, FTIR, or mechanical and functional testing.

For pneumatic-seal diagnostics, EPR is therefore potentially valuable as an early-stage degradation indicator, particularly when combined with methods that assess morphology and functional performance. Its current limitation is the relatively small number of comparative datasets linking EPR parameters directly with friction, leakage, or remaining service life.

### 5.4. Thermal and Dynamic-Mechanical Methods

Thermogravimetric analysis (TGA), differential scanning calorimetry (DSC), and dynamic mechanical analysis (DMA) provide complementary information on thermal stability, phase transitions, segmental mobility, damping, and viscoelastic response. These parameters are important because aging can change seal stiffness and elastic recovery before visible damage develops [7,15].

TGA is useful for comparing degradation onset and thermal stability, whereas DSC can identify changes in thermal transitions. DMA is particularly relevant to dynamic sealing because it characterizes the temperature- and frequency-dependent viscoelastic response of the polymer.

TGA provides information on thermal stability through parameters such as the onset of mass loss, characteristic decomposition temperatures, and residual mass after degradation. Shifts in these parameters after aging may indicate changes in thermal stability, additive loss, or altered degradation pathways. DSC can be used to determine thermal transitions such as the glass-transition temperature and, where relevant, melting or crystallization behavior; changes in these transitions may reflect modifications in chain mobility, phase structure, or crosslink density. DMA provides temperature- and frequency-dependent storage modulus, loss modulus, and damping behavior (tan δ), which are particularly relevant to dynamic seals because they describe changes in stiffness, energy dissipation, and viscoelastic response under cyclic deformation. Aging-induced changes in glass-transition behavior, storage modulus, or damping may therefore indicate progressive loss of compliance and altered sealing response before visible damage develops [7,14,16,38,39,40].

However, these techniques are primarily material-characterization tools. Their practical value increases when results are correlated with compression set, friction, leakage, or cycling performance.

### 5.5. Hardness, Compression Set, and Stress Relaxation

Hardness is frequently used because it is simple and rapid, but it should not be treated as a standalone degradation criterion. Aging may increase hardness while the seal remains functional, or significant loss of sealing capability may occur with only moderate hardness changes [6,7].

Compression set and stress relaxation are more directly related to the ability of an elastomeric seal to maintain contact pressure. Long-term studies of HNBR, EPDM, and FKM have shown that these parameters are valuable for lifetime assessment, particularly when combined with leakage measurements [6,7,14].

Thus, compression set is best interpreted as an indicator of loss of elastic recovery, not as a universal end-of-life threshold.

### 5.6. Surface Profilometry and Tribological Testing

Surface profilometry can quantify roughness evolution, wear-track geometry, and local material loss. These parameters are particularly useful for comparing new and aged seals or for evaluating the influence of different counterface conditions.

Tribological testing provides direct information on friction coefficient, wear rate, breakaway friction, and transitions between sliding regimes. For pneumatic applications, this is essential because increased friction may degrade actuator performance before leakage occurs [2,4].

The strongest experimental protocols therefore combine material-state characterization with tribological response. For example, SEM or FTIR can indicate why a surface has degraded, whereas friction measurements show the functional consequence of that degradation.

### 5.7. Integrated Diagnostic Assessment

**Figure 2:**
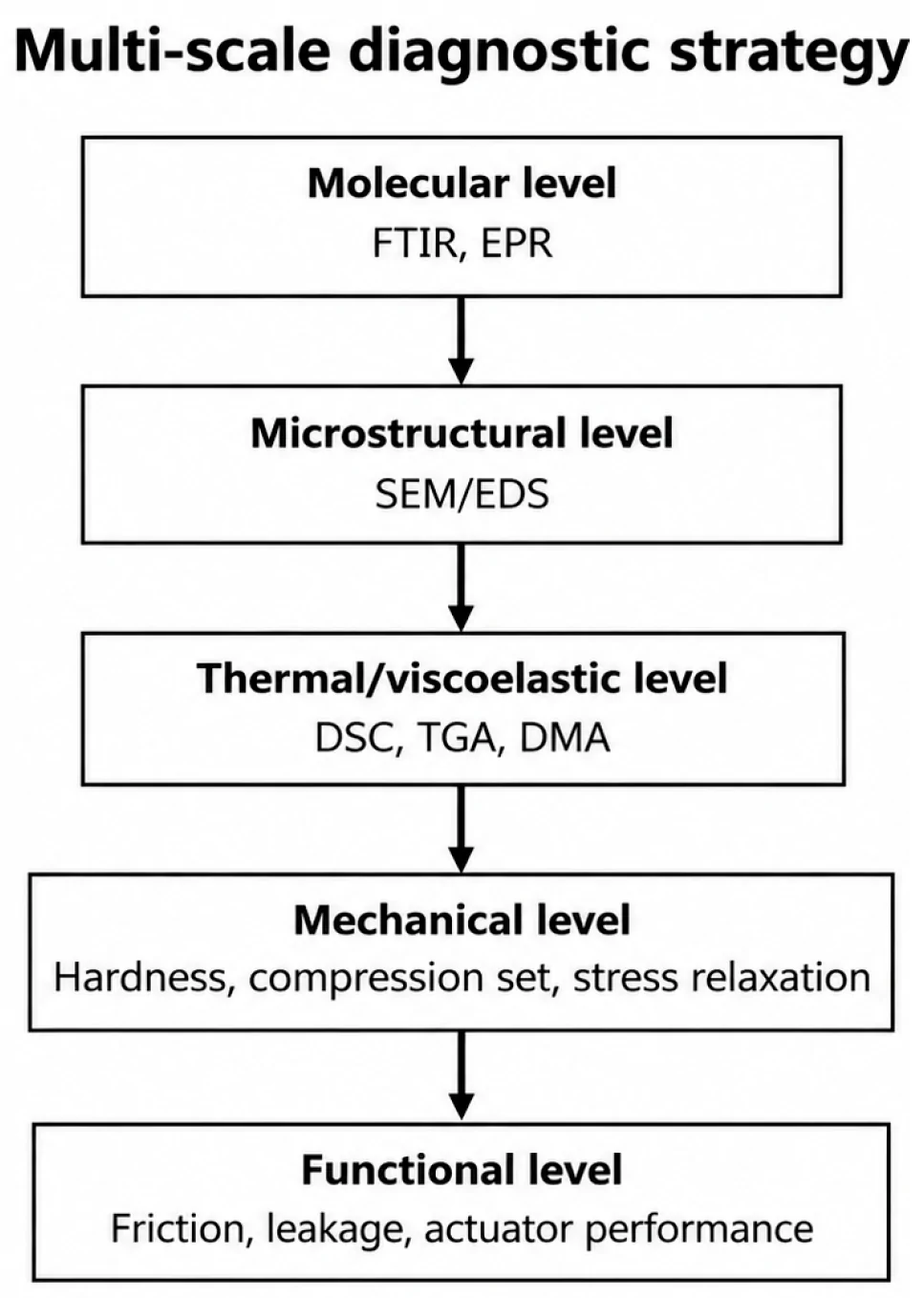
Multi-scale diagnostic strategy for the characterization of degradation in polymeric sealing materials, combining molecular, microstructural, thermal, viscoelastic, mechanical, and functional assessment methods.

The principal limitation of current seal-diagnostics research is the frequent use of isolated characterization methods. A single technique may detect degradation without showing whether the seal has actually lost functionality. To integrate diagnostic information obtained at different scales, a multi-scale characterization strategy is proposed, as illustrated in Figure 2.

Such an integrated approach is particularly important for pneumatic seals because the transition from material degradation to functional failure may be gradual and strongly dependent on seal geometry and operating conditions. The principal diagnostic and characterization methods used to assess degradation of polymeric sealing materials are summarized in Table 4.

## 6. Accelerated Aging and Service-Life Prediction

Accelerated aging is widely used to estimate the durability of polymeric seals within practical laboratory timescales. The most common approaches include Arrhenius-based extrapolation, time–temperature superposition, and end-of-life criteria based on compression set, stress relaxation, or leakage [6,7,13,14].

Arrhenius-based methods assume that elevated temperature accelerates the same degradation mechanisms that occur during normal service. This approach is useful when the degradation pathway remains unchanged, but excessively high temperatures may activate additional reactions and lead to inaccurate lifetime estimates [7,13]. Time–temperature superposition provides an alternative for viscoelastic materials and has been successfully applied to long-term compression-set and stress-relaxation behavior [7,14].

In time–temperature superposition, degradation or viscoelastic-response curves obtained at different temperatures are shifted horizontally along the logarithmic time axis to a selected reference temperature. The corresponding shift factor, ***a_T_***, represents the relative acceleration or deceleration of the process at a given temperature. By applying these shift factors, individual compression-set or stress-relaxation curves can be combined into a single master curve covering a substantially longer effective time range than that accessible experimentally at one temperature. The temperature dependence of ***a_T_*** may subsequently be described using Arrhenius-type or other empirical relationships. The validity of the resulting master curve nevertheless depends on thermorheological or mechanistic simplicity; if aging temperature changes the dominant degradation mechanism, network structure, or oxidation regime, the individual curves may not be superposable and the extrapolation may become unreliable [7,14,41,42].

A critical issue is the definition of end-of-life. Changes in hardness, tensile properties, or compression set do not necessarily coincide with functional failure. Long-term O-ring studies have shown that substantial material degradation may occur while sealing capability is still retained [6,7]. Therefore, leakage-based criteria are generally more relevant because they define failure directly in terms of loss of sealing function [14].

For pneumatic actuators, leakage should not be the only functional indicator. Increasing breakaway force, dynamic friction, unstable motion, reduced repeatability, and higher compressed-air consumption may reveal seal deterioration before gross leakage develops.

Reliability models that combine material degradation with variable mechanical loading offer a more realistic approach than deterministic lifetime estimates [43].

Several classes of reliability models can be used for sealing components, depending on the available data and the definition of failure. Time-to-failure data may be described using statistical lifetime distributions such as the Weibull distribution, whereas accelerated-aging data can be combined with temperature-dependent relationships to obtain Arrhenius-based reliability models. Degradation-process approaches use continuously measured indicators, such as compression set or stress relaxation, to estimate the probability that a predefined failure threshold will be exceeded. More advanced time-variant reliability models combine degradation of material properties with variability in mechanical loading and contact stress, thereby accounting for both aging and stochastic operating conditions [43]. Such models generally require substantially more input data than deterministic lifetime extrapolation, including repeated degradation measurements, statistical distributions of loads or material properties, and a clearly defined functional failure criterion. Their predictive quality therefore depends strongly on sample size, parameter uncertainty, and the representativeness of the accelerated test conditions.

However, most current studies still rely on single-stressor aging, whereas pneumatic seals are exposed simultaneously to temperature, friction, humidity, cyclic deformation, lubricants, and contamination [43].

Future lifetime-prediction methods should therefore combine accelerated aging, material characterization, tribological testing, and direct functional measurements. This would allow degradation indicators to be linked with actual seal performance and remaining service life. The main accelerated-aging approaches and service-life prediction methods applicable to polymeric sealing materials are summarized in Table 5.

Additional evidence from tribological and aging studies further supports the need for a multi-factor assessment of sealing materials. Friction in elastomeric pneumatic-cylinder seals is strongly affected by contact conditions and material response [33], while studies on HNBR have shown that lubrication regime, swelling, and hard-particle contamination can substantially modify friction and wear behavior [44,45]. Long-term thermo-oxidative degradation may also be influenced by diffusion-limited oxidation and deviations from simple Arrhenius behavior, particularly during accelerated aging [46,47,48]. Compression stress relaxation and loss of sealing recovery provide additional indicators of degradation in elastomeric seals [49,50]. Recent studies of nitrile-based and other rubber sealing materials have further demonstrated the importance of coupled thermal, oil, chemical, and environmental exposure [51,52,53]. For PTFE-based sealing systems, humidity and counterface material may significantly affect friction, wear, and transfer-film behavior [54].

The Arrhenius approach assumes that the dominant degradation mechanism remains unchanged over the temperature range used for extrapolation and that the temperature dependence of the degradation rate can be represented by a single apparent activation energy. This assumption may not always be valid for sealing elastomers. At elevated temperatures, changes in the balance between chain scission and crosslinking, altered oxygen transport, or diffusion-limited oxidation can modify the apparent degradation kinetics. Under such conditions, a single linear Arrhenius relationship may lead to inaccurate lifetime estimates. Long-term aging studies of elastomeric O-rings have shown that deviations from simple Arrhenius behavior may occur when oxidation becomes diffusion-controlled or when the dominant degradation mechanism changes with temperature [46,47,48,55,56]. Therefore, Arrhenius extrapolation should preferably be supported by sufficiently long aging periods and by verification that the same degradation mechanism is preserved across the investigated temperature range.

## 7. Integrated Material-Selection Framework

The reviewed literature shows that material selection for pneumatic seals should be based on the dominant service stressors rather than on nominal material properties alone. The most relevant factors are wear, temperature, humidity, lubricant exposure, chemical environment, friction requirements, and expected duty cycle [33,44,45,51,52,53,54].

Recent long-term aging studies further indicate that material selection should account not only for nominal chemical resistance but also for the evolution of recovery, stress relaxation, and sealing force during service. EPDM O-rings aged for periods of up to five years exhibited temperature-dependent recovery behavior and changes in leak-tightness, confirming that compression-set data should be interpreted together with functional sealing criteria [57]. Similar conclusions have been obtained from oxidative-aging studies combining experimental characterization with constitutive or degradation modeling [58,59].

This distinction is further supported by studies showing that sealing-force retention, stress relaxation, compression set, and leakage do not evolve identically during aging. Stress-relaxation experiments on elastomeric seals have demonstrated a strong dependence of sealing performance on temperature and relaxation history [49,60], while thermo-oxidative aging of EPDM under compressive stress has shown that changes in crosslink density and network structure can progressively reduce sealing resilience [61].

Compression stress-relaxation studies have shown that high-temperature data may produce misleading lifetime estimates when diffusion-limited oxidation or non-Arrhenius behavior occurs [55,56]. Long-term O-ring investigations have further demonstrated that reliable extrapolation requires sufficiently long aging periods and careful definition of end-of-life criteria [7]. Similar approaches based on compression stress relaxation have also been successfully applied to fluorosilicone seals, confirming the usefulness of sealing-force decay as a lifetime indicator [62]. More recent modeling studies combine stress relaxation, chemical degradation, and time–temperature superposition to improve long-term prediction under thermo-oxidative aging [41,42].

Recent reviews further emphasize that reliable assessment of elastomer degradation requires the integration of chemical, thermal, mechanical, and functional indicators rather than reliance on a single material parameter [63,64]. Dynamic mechanical analysis is particularly useful in this context because aging-induced changes in modulus, damping, and viscoelastic response may provide additional information on the evolution of sealing-material performance under cyclic loading [38].

For conventional reciprocating pneumatic actuators, PU and NBR/HNBR remain the most versatile options. PU is preferable where abrasion resistance dominates, whereas NBR is suitable for moderate-temperature, oil-compatible service. HNBR becomes advantageous when improved thermal and oxidative stability is required. FKM is most appropriate for chemically or thermally demanding environments, while EPDM is better suited to humid or ozone-rich conditions. Silicone is useful where a wide temperature range is critical, and PTFE-based materials are attractive when low friction and chemical inertness are the main design requirements [24,25,26,27,28,29,30,31,32].

Material-specific studies further support this stressor-dependent selection approach. For NBR, combined oil and thermal aging, as well as thermo-oxidative exposure, can significantly alter sealing and tribological performance [65,66]. HNBR provides improved resistance under elevated-temperature service, although long-term thermal aging still affects sealing behavior [67]. FKM remains advantageous in demanding thermal and chemical environments [68]. PTFE-based materials are particularly attractive where low friction and wear control are critical [69,70], while polyurethane shows strong resistance to severe frictional and wear loading [71]. EPDM is advantageous under environmental exposure involving moisture and aging [72], whereas silicone elastomers retain useful properties over broad thermal and environmental conditions but may undergo significant surface and micro-contact degradation [73]. Table 6 summarizes a simplified material-selection framework for pneumatic sealing systems.

## 8. Discussion

The literature shows that the long-term performance of pneumatic seals cannot be explained by polymer type alone. Functional durability results from the interaction between material chemistry, seal geometry, operating conditions, tribological contact, and degradation history. Although PU, NBR/HNBR, FKM, EPDM, silicone, and PTFE-based materials exhibit distinct advantages, none provides universally superior performance under all pneumatic service conditions [1,2,4,11].

In pneumatic sealing systems, functional performance is also strongly governed by the contact mechanics at the seal–counterface interface. Experimental and numerical studies have shown that contact pressure distribution directly affects both sealing capability and friction, while operating pressure, lubrication conditions, material response, and sliding velocity modify the resulting tribological behavior [74,75]. More recent experimental work on pneumatic rod seals further confirmed that contact pressure cannot be treated as a purely geometric parameter, because it evolves with loading and directly influences the balance between leakage prevention and frictional losses [76].

One of the clearest conclusions is that material degradation and functional failure are not equivalent phenomena. Changes in hardness, crosslink density, compression set, morphology, or chemical structure may become measurable before leakage develops, whereas changes in friction can already affect actuator behavior while the seal remains nominally tight [4,5,6,7]. This distinction is important because many conventional aging studies define end-of-life using an arbitrary material-property threshold rather than actual sealing performance.

This discrepancy has also been demonstrated experimentally for elastomeric O-rings, where substantial changes in hardness, compression set, and stress relaxation may occur while the seal remains leak-tight under static conditions [77]. Consequently, leakage alone may be an insufficient early indicator of degradation, particularly when adhesion, residual contact pressure, or heterogeneous oxidation temporarily preserve sealing capability [77,78]. Long-term lifetime studies further show that reliable service-life prediction requires correlation between material-property evolution, sealing-force retention, and actual functional failure, rather than reliance on a single threshold parameter [7].

The available studies also show that degradation mechanisms are strongly coupled. Thermo-oxidative aging may increase stiffness and reduce elastic recovery; frictional heating can accelerate chemical degradation; hydrothermal exposure can change both polymer-network structure and surface morphology; and contamination can intensify wear after the surface has already been weakened by aging [4,5,11,15,17]. Consequently, single-stressor accelerated-aging tests provide only a partial representation of real pneumatic service.

This interpretation is supported by tribological studies showing that thermo-oxidative aging can substantially modify the mechanical and frictional response of rubber sealing materials and can shift the dominant wear mechanism from adhesive toward abrasive or fatigue-related damage [79]. Moreover, recent investigations have demonstrated that specimen geometry and oxygen transport may significantly affect the apparent kinetics of thermo-oxidative degradation in NBR and EPDM, emphasizing the need for caution when transferring accelerated-aging results to real sealing components [80,81].

Another important observation concerns the transferability of data from hydraulic and static sealing systems. Such studies provide valuable information on polymer degradation, compression set, tribology, and lifetime prediction [1,5,6,7,11,14], but pneumatic actuators differ in working medium, lubrication regime, pressure level, and interfacial conditions. Therefore, hydraulic and O-ring data should support mechanistic interpretation rather than be treated as direct substitutes for pneumatic-specific endurance data.

Pneumatic sealing systems require particular attention because the frictional response of the seal may differ substantially from that observed under fully lubricated hydraulic conditions. Dedicated pneumatic test rigs have shown that the contribution of individual piston and rod seals to the total cylinder friction can be measured separately and that seal friction depends strongly on the specific component configuration and operating conditions [34]. Surface engineering of elastomeric seals has also been investigated directly in pneumatic actuators, demonstrating that modification of the seal–counterface interaction can significantly alter frictional behavior [35,36].

The literature also indicates that multi-method diagnostics are more informative than isolated measurements. SEM/EDS, FTIR, EPR, thermal analysis, hardness, compression set, stress relaxation, and tribological testing describe different stages of degradation [4,5,12,14]. Their combined use can potentially distinguish early physicochemical changes from advanced functional deterioration.

Recent studies confirm that combining spectroscopic, microscopic, thermal, and mechanical methods provides a more complete description of seal degradation than any single technique used in isolation. In situ FTIR has been shown to detect early thermo-oxidative changes in NBR and to correlate spectroscopic indicators with mechanical-property evolution [37]. Multi-method studies of aged rubber seals have additionally demonstrated that SEM, FTIR, DMA, TGA, hardness, and compression-set measurements can reveal complementary stages of degradation and support service-life assessment [39,40].

In particular, EPR applied to polyurethane seals from pneumatic drives can help detect service-induced changes that are not captured by conventional mechanical inspection alone [12].

From a materials-selection perspective, the reviewed evidence supports a stressor-dependent approach. PU/TPU is particularly suitable where abrasion resistance dominates; NBR remains attractive under moderate thermal conditions and oil-compatible service; HNBR provides improved thermal and oxidative stability; FKM is preferable in chemically or thermally demanding environments; EPDM is useful under humid and ozone-rich conditions; silicone is advantageous over wide temperature ranges; and PTFE-based systems are relevant where low friction and chemical inertness are critical [1,4,5,11,14,27,28,30,32].

A key research need is not simply to identify “better” sealing polymers, but to establish quantitative relationships between material degradation state and functional pneumatic performance. Future studies should combine representative pneumatic cycling with controlled thermal, humidity, lubrication, and contamination conditions and should simultaneously monitor friction, leakage, actuator repeatability, surface morphology, and physicochemical degradation. Such data would provide a basis for degradation-state models and remaining-service-life prediction.

Data-driven prognostic approaches may further complement conventional accelerated-aging models. Hybrid machine-learning methods have already been applied to reciprocating-seal degradation prediction, demonstrating the feasibility of forecasting degradation trends from experimentally acquired condition data [82].

Such approaches are potentially useful when degradation is governed by several interacting variables and cannot be described adequately by a single physical parameter [82]. Their application requires representative degradation datasets obtained under controlled and well-documented operating conditions. For sealing systems, relevant inputs may include experimentally measured condition indicators related to wear, friction, leakage, or material degradation [82,83]. A major limitation is that models developed using data from a specific experimental configuration may not be directly transferable to different seal geometries, materials, or operating conditions. In addition, the availability of sufficiently long degradation datasets remains an important constraint. Data-driven approaches should therefore be regarded as complementary to physics-based aging and reliability models, rather than as direct replacements for them [82,84].

Reliability-based lifetime models have also been developed for NBR O-rings by jointly considering compression set and compressive stress relaxation, providing a more realistic representation of multi-parameter degradation than single-indicator approaches [84]. In addition, wear-debris characterization has been proposed as a condition-based indicator of seal deterioration, highlighting the potential of tribological monitoring for predictive maintenance of sealing systems [83].

These issues are relevant not only to the sealing element but also to the performance of the entire pneumatic system. Pneumatic systems are among the most widely used actuation technologies in manufacturing, automation, packaging, transport, robotics, and process engineering. Because compressed-air systems are deployed on a very large scale, improvements in seal durability, friction reduction, leakage control, and service-life prediction can improve higher energy efficiency, lower maintenance costs, improved production continuity, and greater system reliability [85,86]. Therefore, understanding and controlling polymeric seal degradation is not only a materials-science problem but also an important industrial and economic challenge.

## 9. Conclusions

The main conclusions of this review can be summarized as follows:

1. No single polymeric sealing material is universally optimal for pneumatic actuators. PU/TPU is particularly advantageous where abrasion resistance is critical; NBR and HNBR are suitable for oil-compatible service; FKM is preferable under severe thermal and chemical exposure; EPDM and silicone are better suited to specific environmental conditions; and PTFE-based materials are attractive where low friction is a primary requirement.
2. Seal durability is governed by coupled degradation mechanisms rather than by a single operating stressor. Thermal aging, friction, humidity, lubricant interaction, cyclic deformation, contamination, and contact conditions may interact and accelerate material deterioration.
3. Material degradation and functional seal failure should be treated as related but distinct phenomena. Changes in hardness, compression set, stress relaxation, surface morphology, or chemical structure may occur well before measurable leakage or complete loss of sealing capability.
4. Reliable degradation assessment requires a multi-method diagnostic strategy. Spectroscopic, microscopic, thermal, mechanical, and tribological techniques should be combined with direct functional measurements such as friction, leakage, breakaway force, and actuator repeatability.
5. Future service-life prediction should move beyond single-parameter accelerated-aging models. The most promising approach is to integrate representative pneumatic cycling, degradation-state diagnostics, functional performance measurements, and reliability or data-driven prognostic methods to estimate remaining service life.

## Figures and Tables

**Table 1:** Comparative properties, degradation mechanisms, and application suitability of polymeric sealing materials relevant to pneumatic actuators.

Material	Principal Advantages	Main Limitations	Degradation and Damage Pathway	Particularly Suitable Conditions
PU/TPU	High abrasion resistance; good load-bearing capability; good elastic response [1,2,12,15]	Hydrolysis sensitivity in some formulations; frictional heating [12,15]	Hydrolysis and thermo-mechanical degradation; fatigue, abrasion, and cracking [12,15]	Dynamic reciprocating seals where wear resistance is critical [2]
NBR	Good oil resistance; low cost; good sealing elasticity [1,17]	Lower thermo-oxidative stability; humidity and oil-aging sensitivity [4,17]	Thermo-oxidative and hydrothermal degradation, crosslinking, chain scission, and additive migration; surface microporosity, hardening, and compression set [4,17]	Moderate-temperature, oil-compatible pneumatic systems [1,4]
HNBR	Better thermal and oxidative stability than NBR [5]	Higher cost; aging under compression and elevated temperature [5]	Thermo-oxidative degradation, crosslinking, and chain scission; hardening, compression set, and loss of elastic recovery [5]	More demanding thermal or chemically exposed systems [5]
FKM	Excellent thermal and chemical resistance [11,20]	Higher cost; lower low-temperature flexibility; hardening during aging [20]	Dehydrofluorination, chain scission, and thermo-oxidative degradation; hardening and abrasive surface damage [11,18,20]	High-temperature or chemically aggressive service [20]
EPDM	Excellent resistance to water, steam, ozone, and weathering [21,22]	Poor compatibility with many mineral oils [21,22]	Thermo-oxidative degradation and changes in crosslink density; compression set, hardening, and dimensional changes [21,22]	Humid, aqueous, outdoor, or ozone-exposed environments [21]
Silicone	Wide service-temperature range; good low-temperature flexibility [24,25]	Lower abrasion and tear resistance [24,25]	Thermal aging and network degradation; hardening, compression set, and mechanical wear [24,25]	Low-load seals exposed to wide temperature variations [24,25]
PTFE/filled PTFE	Very low friction; excellent chemical resistance [27,28,30,32]	Creep; wear of unfilled grades; limited elastic recovery [27,28,30,32]	Creep and tribological degradation, including transfer-film instability; wear and permanent deformation [27,28,30,32]	Low-friction and chemically aggressive environments [27,28,30,32]

**Table 2:** Principal operating stressors and their effects on pneumatic sealing materials.

Operating Stressor or Exposure	Main Material/Contact Response	Functional Consequence	Materials with Notable Susceptibility	References
Cyclic mechanical loading	Fatigue, permanent deformation, loss of recovery	Reduced contact pressure, leakage	PU, NBR, HNBR	[2,5]
High sliding speed and frictional heating	Local thermal aging, surface softening or hardening, accelerated wear	Increased friction, stick–slip, leakage	PU, NBR, FKM	[4,11,15]
Humidity/condensation	Hydrolysis, additive loss, network changes	Reduced elasticity, dimensional changes, increased friction or leakage	PU, NBR	[4,15]
Incompatible lubricant exposure	Swelling, extraction, additive migration	Altered friction, dimensions, and mechanical properties	NBR, HNBR	[5,17]
Contamination	Abrasive wear, particle embedding	Surface damage, leakage	PU, FKM, PTFE systems	[11,27,28,29,30,31,32]
Misalignment or eccentric loading	Non-uniform contact pressure, asymmetric deformation, localized wear	Increased friction, premature leakage, reduced service life	All dynamic seals	[2]

**Table 3:** Main degradation mechanisms of polymeric sealing materials and their functional consequences.

Degradation Mechanism	Typical Material Changes	Main Functional Consequences	Materials Particularly Affected	References
Thermo-oxidative aging	Oxidation, hardening, network modification	Reduced elasticity, higher compression set	NBR, HNBR, FKM, EPDM	[5,7,11,14,17]
Chain scission	Reduction in molecular integrity, embrittlement	Cracking, loss of strength	NBR, HNBR, FKM, PU	[5,11,14,15]
Additional crosslinking	Increased stiffness and hardness	Reduced recovery, higher friction	NBR, HNBR, EPDM	[4,5,17]
Hydrolysis	Molecular-chain cleavage	Softening, strength loss, surface deterioration	PU/TPU	[15]
Swelling and extraction	Dimensional changes, additive loss	Altered contact pressure and friction	NBR, HNBR	[5,17]
Compression set	Permanent deformation	Reduced sealing force, leakage	Elastomers generally	[6,7,14]
Fatigue and cracking	Microcracks, tearing, surface damage	Leakage and premature failure	PU, NBR, HNBR	[2,15]
Tribological wear	Abrasion, debris, material removal	Friction increase, leakage	PU, FKM, PTFE systems	[2,11,27,28,29,30,32]

**Table 4:** Diagnostic and characterization methods for evaluating degradation of polymeric sealing materials and their relevance to seal performance assessment.

Method	Main Information Obtained	Main Advantage	Main Limitation	References
SEM	Cracks, pores, wear scars, debris, fracture morphology	Direct visualization of surface damage	Mainly morphological information	[4,5,12]
EDS	Elemental composition, contamination, transferred particles	Identifies inorganic inclusions and transfer	Limited information on polymer chemistry	[12]
FTIR	Oxidation, hydrolysis, functional-group changes	Sensitive to chemical degradation	Weak direct link with sealing function	[5,14,15]
EPR	Radicals and paramagnetic centers	Sensitive to selected degradation processes	Limited seal-specific datasets	[12]
TGA/DSC	Thermal stability and transitions	Useful for comparing aging states	Indirect relation to leakage	[11,13]
DMA	Viscoelastic response, modulus, damping	Relevant to cyclic sealing behavior	Requires interpretation under representative conditions	[14,15]
Hardness	Surface/bulk stiffness changes	Rapid and simple	Poor standalone end-of-life criterion	[6,7]
Compression set	Loss of elastic recovery	Closely related to sealing-force retention	Geometry-dependent	[6,7,14]
Stress relaxation	Reduction in contact force with time	Directly relevant to static sealing force	Less commonly measured in pneumatic studies	[7,14]
Profilometry	Roughness and wear geometry	Quantifies surface evolution	Does not identify chemical mechanism	[2,11]
Tribological testing	Friction, wear, breakaway behavior	Direct functional relevance	Highly condition-dependent	[2,4,11,27,28,30,32]
Leakage testing	Direct loss of sealing function	Closest to actual end-of-life	Does not identify degradation mechanism	[6,7,14]

**Table 5:** Accelerated-aging approaches and service-life prediction methods for polymeric sealing materials.

Approach	Main Output	Main Limitation	References
Arrhenius extrapolation	Temperature-dependent lifetime	Requires unchanged degradation mechanism	[7,13]
Time–temperature superposition	Long-term aging curve	Sensitive to mechanism changes	[7,14]
Compression set, stress relaxation	Loss of elastic recovery	Threshold depends on seal geometry	[6,7,14]
Leakage criterion	Functional end-of-life	Does not identify degradation mechanism	[6,14]
Reliability modeling	Probability of failure over time	Requires extensive input data	[43]

**Table 6:** Simplified material-selection framework for pneumatic sealing systems.

Operating Requirement	Preferred Material	References
High abrasion resistance	PU/TPU	[2,12,15,71]
Moderate temperature + oil exposure	NBR	[65,66]
Higher thermal/oxidative stability	HNBR	[67]
High temperature/aggressive chemicals	FKM	[68]
Humid/ozone-rich environment	EPDM	[21,22,72]
Wide temperature range	Silicone	[24,25,73]
Low friction/chemical inertness	PTFE-based materials	[69,70]

## Data Availability

No new data were created or analyzed in this study. Data sharing is not applicable to this article.
